# Transient Temperature at Tool–Chip Interface during Initial Period of Chip Formation in Orthogonal Cutting of Inconel 718

**DOI:** 10.3390/ma17102232

**Published:** 2024-05-09

**Authors:** Youssef Alammari, Jian Weng, Jannis Saelzer, Dirk Biermann

**Affiliations:** 1 Department of Mechanical Engineering, College of Engineering, Qassim University, Buraydah 51452, Saudi Arabia; y.alammari@qu.edu.sa; 2 Institute of Machining Technology, TU Dortmund University, Baroper Str. 303, 44227 Dortmund, Germany; jannis.saelzer@tu-dortmund.de (J.S.); dirk.biermann@tu-dortmund.de (D.B.)

**Keywords:** machining, transient thermal modeling, initial period of chip formation, Inconel 718, FEM, metals, modeling

## Abstract

Machining nickel-based super alloys such as Inconel 718 generates a high thermal load induced via friction and plastic deformation, causing these alloys to be among most difficult-to-cut materials. Localized heat generation occurring in machining induces high temperature gradients. Experimental techniques for determining cutting tool temperature are challenging due to the small dimensions of the heat source and the chips produced, making it difficult to observe the tool–chip interface. Therefore, theoretical analysis of cutting temperatures is crucial for understanding heat generation and temperature distribution during cutting operations. Periodic heating and cooling occurring during cutting and interruption, respectively, are modeled using a hybrid analytical and finite element (FE) transient thermal model. In addition to identifying a transition distance associated with initial period of chip formation (IPCF) from apparent coefficient of friction results using a sigmoid function, the transition temperature is also identified using the thermal model. The model is validated experimentally by measuring the tool–chip interface temperature using a two-color pyrometer at a specific cutting distance. Due to the cyclic behavior in interrupted cutting, where a steady-state condition may or may not be achieved, transient thermal modeling is required in this case. Input parameters required to identify the heat flux for the transient thermal model are obtained experimentally and the definitions of heat-flux-reducing factors along the cutting path are associated with interruptions and the repeating IPCF. The thermal model consists of two main parts: one is related to identifying the heat flux, and the other part involves the determination of the temperature field within the tool using a partial differential equation (PDE) solved numerically via a 2D finite element method.

## 1. Introduction

**Figure 1:**
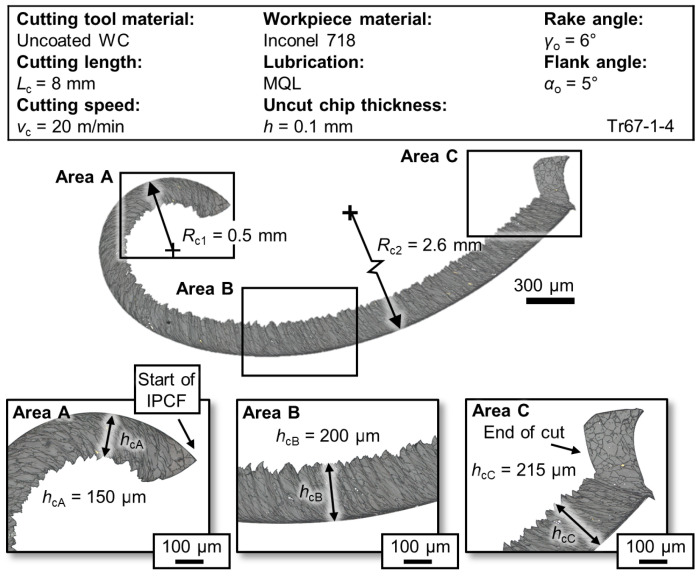
Main characteristics of an IPCF chip—obtained experimentally. Chip thickness *h*_c_ was measured using a calibrated optical microscope. Curl radius *R*_c_ was calculated through fitting an arc of 3 points to the contact side of the chip.

Machining difficult-to-cut materials like Inconel 718 presents significant challenges due to the material’s mechanical and thermal properties, resulting in high mechanical and thermal loads. De Bartolomeis et al. [1] extensively reviewed the machinability of Inconel 718. Typically, machining this alloy involves flood cooling with metalworking fluids (MWF), but sustainable practices suggest minimizing or eliminating MWF use. Recent contributions to the literature [2,3] have explored sustainable machining methods across various materials and processes. The observed low tool–chip contact in the initial period of chip formation (IPCF), where reduced tool–chip friction is observed at the beginning of cutting [4], indicates promising potential for sustainable machining. Figure 1 illustrates the main characteristics of IPCF chips: small chip thickness, large shear angle, minimal secondary shear zone, and small chip curl radius. These characteristics, in addition to reduced passive force, are all indicators of reduced tool–chip contact friction. However, additional process adaptation may be necessary to enable and sustain the effect of the IPCF in continuous machining operations. For example, interruption can be induced using intermittent vibration-assisted machining to influence MWF delivery to the tool–chip interface. To adopt sustainable lubrication like MQL for Inconel 718, it is crucial to fundamentally investigate chip formation and the related thermal effects to understand the tribological aspects involved.

Measuring temperature in machining can be quite challenging, especially within the tool–chip interface, as the temperature distribution changes considerably along the rake face with a steep temperature field. Additionally, the transient behavior occurring in interrupted machining, which lasts for a very short time, poses an additional challenge for experimental temperature investigations. Researchers have experimentally investigated temperature measurement using different techniques, as reviewed in a CIRP keynote paper by Davies et al. [5]. In addition to experimental temperature measurement, analytical and numerical models provide detailed insights into the transient temperature behavior, both spatially and temporally.

Trigger and Chao [6] conducted pioneering research on determining cutting temperatures in continuous cutting. The heat source in the shear plane was accounted for in the model and the average temperature of the chip in the shear zone was evaluated. Other researchers have built upon Trigger and Chao’s model for continuous cutting. Komanduri and Hou [7] investigated the orthogonal continuous cutting process regarding shear plane heat sources. The frictional heat source within the tool–chip interface was incorporated first [8]. Later, the heat from two sources, friction and shearing, was incorporated [9]. Chenwei et al. [10] proposed an enhanced model based on the Komanduri–Hou and Huang–Liang models. To validate their model, they incorporated a thermocouple into the cutting tool during the cutting of a titanium alloy. Weng et al. [11] introduced an enhanced analytical thermal model, building upon the Komanduri–Hou framework, to predict the steady-state temperature of the rake face. Their method incorporated temperature-dependent thermal properties and was validated through direct in situ temperature measurements using a two-color pyrometer. Salame and Malakizadi [12] investigated an improved semi-analytical thermal model, which employed physics-based estimation to account for variable heat flux at the tool–chip interface. Ning and Linag [13] performed a comparative study of three widely recognized analytical thermal models to forecast the orthogonal cutting temperature of AISI 1045. Zhao et al. [14] carried out a thorough review of analytical and numerical methods developed to explore the effects of coatings on cutting temperature. Barzegar and Ozlu [15] employed the finite difference method to investigate the steady-state influence of including the cutting edge radius and the third deformation zone. While most research on cutting temperature has focused on continuous cutting operations, several studies have also been conducted on interrupted cutting operations.

In interrupted machining, transient temperature modeling is highly relevant. Only a few researchers have addressed this problem and developed complete analytical or numerical models with experimental validation. Interrupted cutting is characterized by the production of discontinuous chips and cyclic mechanical and thermal loading. The tool and the workpiece are influenced by this type of loading.

Stephenson and Ali [16] utilized an approach where they approximated the tool’s geometry by employing a semi-infinite rectangular corner. This method was employed to simulate the transient temperatures at specific points within the tool during interrupted cutting. They investigated the effects of various heat source distributions across the rake face, along with time-dependent variations in heat source intensities. Their model suggested that interrupted cutting leads to lower tool temperatures compared to continuous cutting due to the cooling effect during non-cutting periods. They explained how the Green’s function can be implemented to solve for temperature within the tool. Identifying the input heat flux can pose challenges, as it requires determining the spatial and temporal distribution of heat flux. Stephenson and Ali utilized the steady-state cutting temperature model developed by Loewen and Shaw [17], which involves calculating the total amount of frictional energy dissipated at the tool–chip interface, based on parameters related to machining process parameters, the mechanical load, and chip-related parameters such as chip velocity, chip thickness, and contact length. The analytical model by Stephenson and Ali [16] is a fundamental approach in transient temperature modeling that has been accepted and implemented by many researchers, such as Karaguzel et al. [18] and Augspurger et al. [19].

Non-uniform heat flux distribution was investigated by Jen and Anagonye [20]. They investigated the influence of initial transient behavior on tool temperature, extending the model of Stephenson and Ali [16]. Jen et al. [21] developed conduction equations in three-dimensional space to represent nonlinear transient heat sources. The equations were numerically solved to study their effect on transient temperature. Potdar and Zehnder [22] developed a finite element model for investigating transient temperature behavior based on a friction model with critical stress criteria. A numerical model was developed by Lazoglu and Altintas [23] to investigate transient temperature fields based on finite difference. Islam et al. [24] developed their work further to include three-dimensional effects. Islam and Altintas [25] used the finite difference method in 2D to investigate the transient conditions related to thermal modelling of coated tools.

Jiang et al. [26] used an analytical model for contact length to determine the time-dependent spatial heat source distribution on the rake face during the milling process. They validated their approach by integrating thermocouples into the tool and used a least square optimization algorithm proposed by Beck et al. [27] to solve the inverse heat conduction problems and determine the heat flux in the tool and workpiece. Liu et al. [28] investigated a three-dimensional analytical model that incorporates transient behavior, including convective cooling, by employing the transient Green’s function method to solve the energy equation. They applied initial and boundary conditions with a defined heat source within the tool–chip interface. To validate the results, a ratio pyrometer was embedded in the cutting tool and positioned at 0.4 mm from the tool tip.

To accurately model cutting temperatures, it is important to consider the contact properties between the cutting tool and chip, particularly the complex frictional interactions at the tool–chip interface. Previous studies have used simplified friction models, such as Coulomb friction. However, Zorev [29] showed that the tool–chip interface has two distinguishable regions: the sticking region and the sliding region. Nevertheless, this distinction is not quite accurate for precise modeling since friction properties also depend on temperature, pressure, and sliding velocity.

Recently, Karaguzel [30] proposed a hybrid model that combines analytical and numerical methods, taking into consideration zonal contact as described by Zorev [29]. The model determines heat flux analytically and uses a numerical transient heat conduction model to calculate temperature within the tool. The MATLAB PDE Toolbox was used to develop the numerical model. A similar approach is utilized in this research for thermal modeling related to IPCF, but with variations in how the heat flux is defined spatially and temporally based on experimental data.

A transition temperature related to the end of the IPCF is obtained by correlating the transition distance with the calculated temperature from the model at the transition distance. Identifying the transition temperature, using the proposed model, enables better understanding of the IPCF, where low friction at the tool–chip interface is observed up to the transition distance. Being able to identify the transition temperature and transition distance facilitates identifying key parameters such as interruption/cutting periods to sustain the favorable effects of the IPCF through interrupted machining.

## 2. Materials and Methods

This section details the experimental work, and a description of the model is provided. Input parameters required in the model are determined experimentally and model results are validated using two-color pyrometer in orthogonal cutting. The model is described in detail, highlighting how interruptions and the effect of the initial period of chip formation are incorporated into it.

### 2.1. Experimental Setup

**Figure 2:**
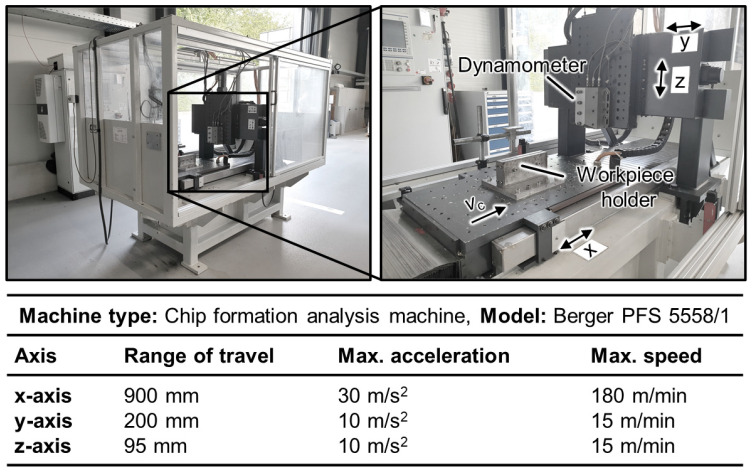
Machine tool used in orthogonal cutting.

A custom-made machine tool was used to conduct orthogonal experiments in the context of this research. The machine used for chip formation analysis was a special machine based on the model PFS 5558/1 from the company Heinz Berger Maschinenfabrik GmbH & Co. KG, Wuppertal, Germany. It has three axes that are utilized for positioning and feed movement. The machine, along with its specifications, is shown in Figure 2.

For the recording of the mechanical tool load, a piezoelectric dynamometer from Kistler Instrumente AG, Winterthur, Switzerland, was used. The force measuring platform, type 9263, offers the possibility to measure mechanical loads of up to 10 kN in the *y*- and *z*-directions and 20 kN in the x-direction. It was attached to a platform sliding on the cross rail of a Berger machine. According to the manufacturer of the dynamometer, its lowest natural frequency is *f*_n_ > 2.5 kHz and stiffness *k*_c_ ≈ 2 kN/μm, with a response threshold of less than 0.1 N. For signal conditioning, Kistler KIAG SWISS, Winterthur, Switzerland, type 5001 charge amplifiers were used to convert the charge signals to proportional voltage signals. The charge amplifiers amplify the signal using calibrated gain factors to within ±10 V. Multi-channel data acquisition from a TEAK Corporation KK, Tokyo, Japan, type GX1 integrated recorder was used to collect the data at a sampling frequency *f*_s_ = 50 kHz upon manual trigger before the beginning of cutting.

To determine the temperature, a two-color pyrometer, Fire III, manufactured by Energy Engineering Aachen (en2AIX) GmbH, Achen, Germany, was used. The pyrometer operates at wavelengths *λ*_1_ = 1.675 μm and *λ*_2_ = 1.945 μm. It has a maximum sampling rate of *f*_s_ = 500 kHz and a temperature range of *T* = 250…1200 °C. To collect the thermal radiation, the pyrometer system utilized a fiber optic cable with a diameter of *d*_fo_ = 330 µm. This fiber optic technology enables measurements in confined spaces.

**Figure 3:**
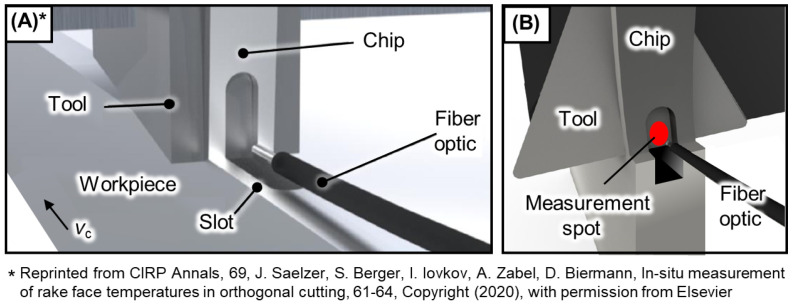
Temperature measurement during orthogonal cutting: (**A**) concept according to Saelzer et al. [33]; (**B**) measurement at the end of the workpiece.

While it is possible to estimate tool temperature using a thermographic camera, as demonstrated by Saez-de-Buruaga et al. [31], this method is subject to fundamental limitations concerning calibration and accuracy, particularly related to emissivity. Additionally, the location from which temperature measurements are typically taken, usually from the tool side, poses challenges [32]. In this study, temperature evaluation utilized a ratio pyrometer to mitigate uncertainties associated with emissivity. The fiber optic of the pyrometer was positioned to directly measure the temperature of the chip-free side. Access to the rake face was facilitated via a small slot along the cutting path, as described by Saelzer et al. [33]. Figure 3 provides an overview of the temperature measurement procedure.

An uncoated tungsten carbide (WC) insert (TPGN160308 H13A) with a cutting edge radius of *r*_β_ = 8 µm and a triangular shape was attached to the tool holder (CTFPL2525M16), both manufactured by Sandvik. The resulting rake angle was *γ*_o_ = 6°, and the clearance angle was *α*_o_ = 5°. Inconel 718, which is the commercial name of the nickel-based superalloy NiCr19Fe19Nb5Mo3 (material number: 2.4886) [34], was used as the workpiece material. It is also known as UNS N07718 according to [35]. The alloy offers high corrosion and creep resistance at elevated temperatures. Due to its desirable thermomechanical properties, it is applied where high temperatures and high mechanical loads occur [1]. For instance, it is used in the hot segments of aero engines and power generation turbines, rocket engine nozzles, nuclear reactors, and in the exhaust systems of high-performance automotive vehicles.

Cuboid workpieces, in the annealed and aged condition with a hardness measured at 470 HV30, were prepared. The workpieces had a width of *b* = 2 mm. The individual cutting lengths *L_ci_* = 8 mm. All experiments were repeated at least twice.

The MQL technique was used to supply lubricants to the cutting zones. The MQL aerosol was prepared remotely using a special aerosol generation device and supplied through a nozzle directed at the cutting zone. The MQL oil employed was Vascomill MMS HD1, a synthetic Ester oil from Blasser Swisslube AG, Rüegsau, Switzerland, consisting of 80% Ester and containing 10% sulfur. This oil has a viscosity of *η* = 40 mm^2^/s at 40 °C and a flash point of *T*_flash_ = 200 °C. The oil was supplied at a flow rate of approximately *Q*_oil_ ≈ 50 mL/h.

### 2.2. Determination of Heat Flux at Tool–Chip Interface

**Figure 4:**
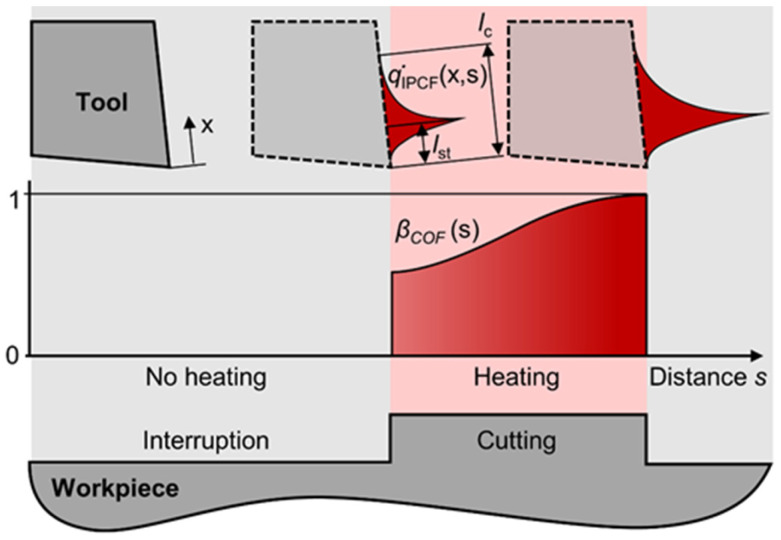
Schematic of heat flux in the IPCF.

In the IPCF, the friction within the tool–chip interface varies over a finite cutting distance, resulting in a corresponding influence on the heat flux. Additionally, the distribution of heat flux within the tool–chip interface is influenced by a dual zone model, namely sticking and sliding. Figure 4 provides an overview of the heat flux within the tool–chip interface in the IPCF.

The primary mechanism in the thermomechanical modeling of the secondary deformation zone is the friction between the tool and the chip. This friction arises from the contact between the two surfaces and is modeled using a dual zone approach [29]. The rake face contact is divided into two distinct regions: a sticking region and a sliding region. In the sticking region, high normal stress occurs, whereas the sliding region exhibits comparatively lower normal stress levels and the Coulomb friction law applies.

Coulomb’s friction law states that the shear stress is directly correlated with the normal stress, governed by sliding friction coefficient *μ* and normal stress *p*. If the friction coefficient remains constant, as the normal stress increases towards the tool tip, the shear stress also increases. Nevertheless, the shear stress cannot surpass the material’s shear flow stress *τ*_1_; thus, it is presumed to be equivalent to *τ*_1_ within the sticking zone. Consequently, the allocation of shear stress across the rake face contact is determined in accordance with Childs [36]:

(1) $$\tau =\left\{\begin{array}{cc} \tau_{1}, & \;\;\;\;x\leq l_{st}\\ \mu p(x), & \;\;\;\;l_{st}<x\leq l_{c} \end{array}\right.$$

Karaguzel [30] provides the expression for the heat flux within the tool–chip interface, represented as $\dot{q}$(*x*), which varies with the distance *x* from the cutting edge on the rake face.

(2) $$\dot{q}\left(x\right)=\left\{\begin{array}{cc} \;\;\tau_{1}v_{ch}, & \;\;\;\;0\leq x\leq l_{st}\\ \mu v_{ch}p(x), & \;\;\;\;l_{st}<x\leq l_{c}\\ \;0, & \;\;\;\;x>l_{c} \end{array}\right.$$

where *l*_c_ represents the total tool–chip contact length, which is measured at the end of adhesion marks appearing on the rake face. *l*_st_ denotes the sticking zone length and is calculated using the formula proposed by Budak and Ozlu [37]:

(3) $$l_{st}=l_{c}\left(-{\left(\frac{\tau_{1}}{p_{0}\mu }\right)}^{\frac{1}{\zeta }}+1\right)$$

where *μ* represents the tool–workpiece sliding friction coefficient, which is determined experimentally. *τ*_1_ represents the shear flow stress of the material, assumed to be equivalent to the shear flow stress in the primary shear zone. This stress is influenced by material properties under conditions of high strain rates and elevated temperatures. It is calculated at as a steady state from measured forces and geometrical parameters, which identifies the primary shearing area, as follows, according to Childs [36]:

(4) $$\tau_{1}=\frac{{(F}_{c}\cos\phi -F_{p}\sin\phi )\cdot \sin(\phi )}{hb}$$

where *F*_c_ represents the cutting force and *F*_p_ denotes the passive force. The angle *ϕ* represents the shear angle, while the uncut chip thickness and width of cut are denoted as *h* and *b*, respectively. *v*_ch_ denotes the chip velocity calculated along the tool–chip contact, following the method proposed by Li et al. [38]:

(5) $$v_{ch}\left(x\right)=\left\{\begin{array}{cc} v_{ch0}{\left(\frac{x}{l_{st}}\right)}^{\omega_{c}}, & \;\;\;\;0\leq x\leq l_{st}\\ v_{ch0}, & \;\;\;\;l_{st}<x\leq l_{c}\\ 0, & \;\;\;\;x>l_{c} \end{array}\right.$$

where *ω*_c_ represents the chip velocity distribution exponent, which is assumed to have a constant value of *ω*_c_ = 2 in this investigation. *v*_ch0_ denotes the average chip velocity and can be determined using the following equation:

(6) $$v_{ch0}=v_{c}\frac{\sin\phi }{cos(\phi -\gamma_{o})}$$

where *v*_c_ represents the cutting speed, *γ*_o_ is the rake angle, and *ϕ* denotes the shear angle, which can be determined geometrically by knowing the chip thickness ratio *r*_c_ = *h*/*h*_c_, where *h*_c_ represents the average chip thickness. The relationship is as follows:

(7) $$\phi =\tan^{-1}\left(\frac{r_{c}\cos\gamma_{o}}{1-r_{c}\sin\gamma_{o}}\right)$$

*p*(*x*) represents the normal pressure on the rake face and is given by Budak and Ozlu [37]:

(8) $$p\left(x\right)=p_{0}{\left(1-\frac{x}{l_{c}}\right)}^{\zeta }$$

Here, *ζ* indicates the stress distribution exponent, usually assumed to range between 2 and 3, and its value can be established via split tool experiments. In the current investigation, it was assumed to be *ζ* = 2. *p*_0_ is calculated according to Budak and Ozlu [37]:

(9) $$p_{0}=\tau_{1}\frac{h(\zeta +1)}{l_{c}sin\phi }\frac{\cos\lambda_{f}}{cos(\phi +\lambda_{f}-\gamma_{o})}$$

where *λ*_f_ represents the apparent friction angle, which is calculated as $\lambda_{f}=\tan^{-1}\left(COF\right)$, and *COF* denotes the apparent coefficient of friction obtained from the cutting forces *F*_c_ and passive forces *F*_p_, as well as the rake angle, using the following equation:

(10) $$COF=\frac{F_{p}+F_{c}\tan\gamma_{o}}{F_{c}-F_{p}\tan\gamma_{o}}$$

The partition ratio *R*_2_ for the secondary shear zone heat flux is employed to determine how much of the overall heat flux $\dot{q}$ is allocated between the tool and the chip. In this investigation, *R*_2_ was found iteratively by comparing the steady-state temperature obtained from the temperature analysis of Loewen and Shaw [17] with the steady-state tool temperature obtained from the proposed model. The tool heat flux was then determined as follows:

(11) $${\dot{q}}_{tool}(x)=\left(1-R_{2}\right)\dot{q}(x)$$

Here, ${\dot{q}}_{tool}$ represents the steady-state heat flux at the tool. The cutting-distance-dependent heat flux in the IPCF was determined both during interruption and cutting as follows:

(12) $${\dot{q}}_{IPCF}\left(x,s\right)=\left\{\begin{array}{cc} {\beta_{COF}\left(s\right)\cdot \dot{q}}_{tool}\left(x\right), & \;\;\;\;i\left(L_{ci}+L_{int}\right)\leq s\leq i\left(L_{ci}+L_{int}\right)+L_{ci}\\ 0, & \;\;\;\;i\left(L_{ci}+L_{int}\right)+L_{ci}\leq s\leq (i+1)\left(L_{ci}+L_{int}\right) \end{array}\right.$$

where *i* = 0, 1, 2, …, *L*_ci_ represents the length of each individual cutting interval, and *L*_int_ denotes the length of the interruption. The distance is related to time by *s* = *v*_c_ *t. β*_COF_ (*s*) is a factor that represents the reduction of steady-state heat flux due to a decrease in friction in the IPCF. It is obtained by fitting a sigmoid function to the average *COF* data of subsequent cutting segments. The sigmoid function for the IPCF is defined as follows:

(13) $$COF_{fit}\left(s\right)={COF}_{min}+\frac{{COF}_{max}}{1+e^{-k_{l}\left(s-s_{0}\right)}},$$

(14) $$\beta_{COF}\left(s\right)=COF_{fit}(s)/{COF}_{max}$$

where *COF*_min_ and *COF*_max_ represent the minimum and maximum *COF* values in the IPCF, respectively. *k*_l_ denotes the evolution rate, and *s*_0_ represents the transition distance at the transition midpoint. If *s*_0_ becomes negative, it indicates that the transition in the *COF* data has not been detected.

### 2.3. Determination of Tool Temperature Due to Heat Flux Occurring at Tool–Chip Interface

Once the heat flux is determined temporally and spatially, as discussed in the previous section, the temperature can be estimated using the heat equation. The partial differential equation for the temperature field in the tool is given as follows:

(15) $$\rho c\frac{\partial T}{\partial t}=\nabla \cdot \left(k\nabla T\right)$$

where *T* represents the tool rake temperature, *ρ* is the density, *k* is the thermal conductivity, and *c* is the specific heat. Equation (15) is solved numerically using the MATLAB 2023a Partial Differential Equations (PDE) toolbox. The two-dimensional tool geometry is specified, and boundary conditions are defined on the edges of the tool as follows:

- Heat flux is applied only within tool–chip contact;
- Heat convection is implemented on both the flank face and the remaining portion of the rake face. The heat convection coefficient is assumed to be *h*_conv_ = 10 W/m^2^, and the ambient temperature is *T*_∞_ = *T*_room_ = 23 °C;
- The outermost edges of the tool are set to a constant temperature of *T*_room_ = 23 °C.

**Figure 5:**
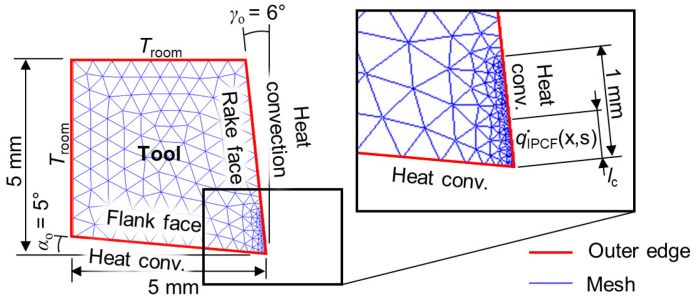
Tool geometry, mesh, and boundary conditions applied in MATLAB PDE toolbox.

Figure 5 provides a summary of the tool geometry, mesh, and boundary conditions of the setup as applied in the MATLAB PDE toolbox. The “generateMesh” command in the MATLAB PDE toolbox was used with *H*_max_ and *H*_edge_ properties.

A refined mesh size with an element length smaller than *L*_elem_ = 0.02 mm was used along the tool–chip contact, extending up to 1 mm from the cutting edge. The maximum element size was *L*_elem_max_ = 0.5 mm throughout the tool, except for the tool–chip contact. The total simulation time was set according to the desired number of heat cycles. The step size was defined by dividing the time of a single heat cycle by 1000.

The partial differential equation was solved using temperature-dependent values of thermal conductivity and specific heat for the tool. They were implemented in the MATLAB PDE toolbox as user defined functions using function handles. In order to determine the heat partition ratio, *R*_2_, temperature-dependent thermal properties for both the tool and workpiece were employed in the model proposed by Loewen and Shaw [17]. Table 1 presents a summary of the temperature-dependent thermal properties for both the tool and the workpieces.

## 3. Results

### 3.1. Input Parameters

**Figure 6:**
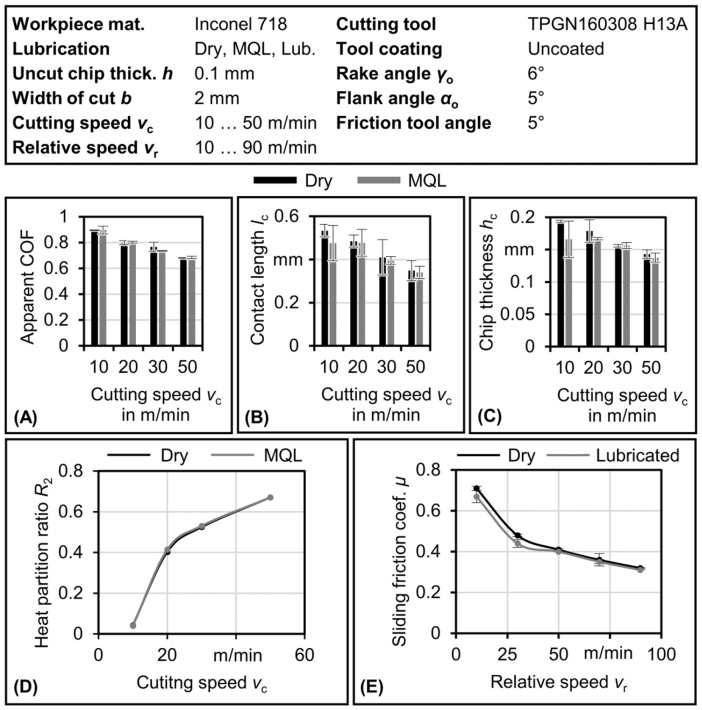
Input parameters of the IPCF transient temperature model for dry and lubricated cases for Inconel 718. (**A**) Apparent friction coefficient (*COF*). (**B**) Tool–chip contact length *l*_c_. (**C**) Chip thickness *h*_c_. (**D**) Heat partition ratio *R*_2_. (**E**) Sliding friction coefficient *μ*.

The model requires certain parameters as inputs. These parameters consist of the apparent coefficient of friction (*COF*), tool–chip contact length at different cutting speeds, mean chip thickness, and the sliding friction coefficient in both dry and lubricated conditions. The sliding friction coefficient was experimentally measured using an open tribometer, as described by Puls et al. [41]. Figure 6 presents a summary of these experimental results. Furthermore, the heat partition ratio *R*_2_, obtained through iterative calculations, is also illustrated in Figure 6.

The parameters shown in Figure 6 are obtained under steady-state conditions. The apparent coefficient of friction influences the heat flux as it defines the maximum normal stress on the rake face, as shown in Equation (8). The contact length is an important factor that determines the spatial extent of the heat flux and is used to obtain the heat partition ratio in the steady state. The contact length impacts the heat partition [42]. The chip thickness and uncut chip thickness are used to calculate the shear angle, considering the rake angle, as shown in Equation (7). The experimentally obtained sliding friction values are used to define the sliding friction in the sliding zone. As expected, the apparent coefficient of friction, tool–chip contact length, and chip thickness decrease as the cutting speed increases. Furthermore, the use of MQL slightly reduces their values. As the cutting speed rises, the heat partition ratio *R*_2_, representing the fraction of heat transferred into the chip, also increases. An interesting observation is the minimal influence of lubrication on the sliding friction for Inconel 718, as shown in Figure 6E.

### 3.2. Validation at Steady State

**Figure 7:**
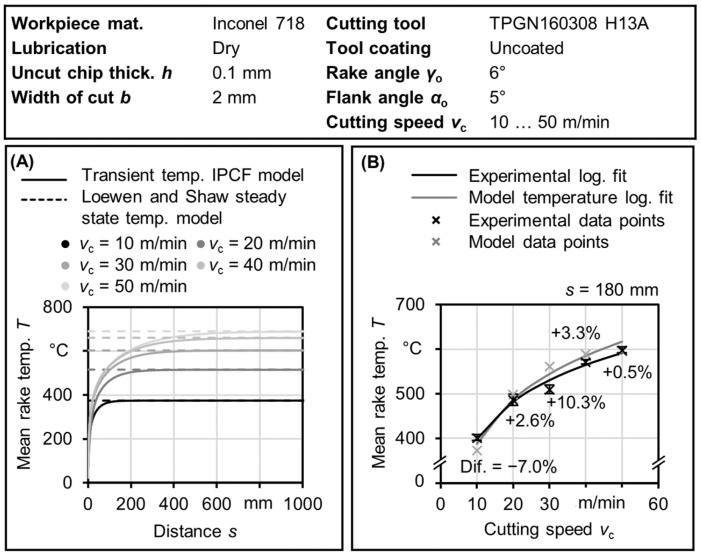
(**A**) Comparison with Loewen and Shaw analytical steady-state model. (**B**) Model results in comparison with experimental average rake temperatures obtained using ratio pyrometer at cutting distance *s* = 180 mm.

The reliability of the proposed model in estimating the steady-state temperature is examined by comparing these results with the results of steady-state temperature model of Loewen and Shaw [17]. Figure 7A provides a summary of the steady-state results. Additionally, a comparison with experimental temperature measurements at a finite distance using a two-color ratio pyrometer on the rake face, as described by Saelzer et al. [33], is shown in Figure 7B.

The results presented in Figure 7 demonstrate the validity of the model. As shown in Figure 7A, the transient behavior exists up to certain distances for different cutting speeds and eventually reaches a steady state. As shown in Figure 7B, the model predicts the average rake temperature, which is the average temperature along the tool chip contact length, with reasonable accuracy. The estimated mean rake temperature error is approximately ±10% within the cutting speed range of *v*_c_ = 10 to 50 m/min for Inconel 718. It is expected that the temperature increases when the cutting speed increases. The model can represent this fundamental phenomenon, which indicates its validity. This error could be attributed to measurement accuracy issues related to the measuring spot size and its exact position on the rake face, as well as inherent limitations of the model resulting from the calculated sticking zone length using Equation (3).

### 3.3. Determination of Heat Flux Reduction Factor within the IPCF

**Figure 8:**
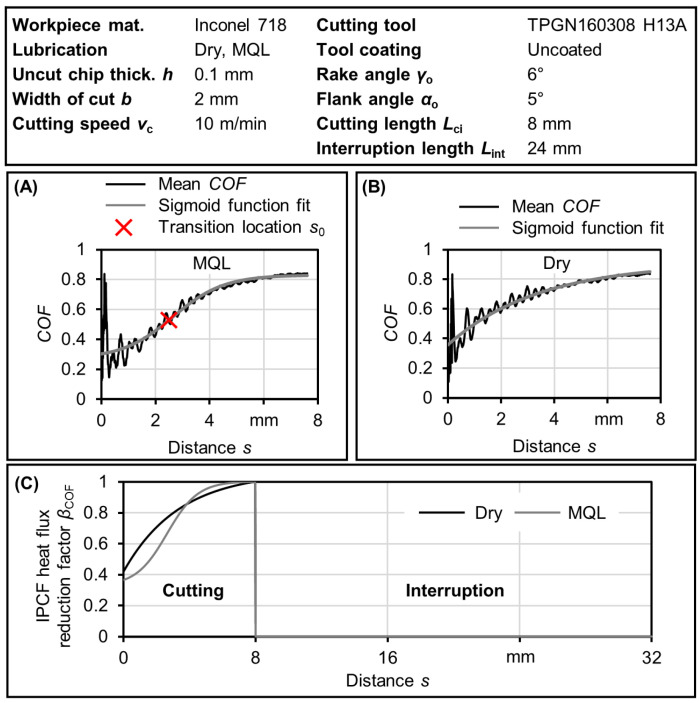
Example of the influence of the IPCF on the heat flux. (**A**) An MQL case showing mean *COF* fitted with sigmoid function with identified transition location *s*_0_. (**B**) Corresponding dry case showing mean *COF* fitted with sigmoid function and no transition in *COF* was identified. (**C**) The resulting IPCF heat flux reduction factor for a single heating cycle of cases shown in (**A**,**B**).

The steady-state heat flux is reduced within the IPCF by utilizing the IPCF heat flux reduction factor *β*_COF_, as shown in Figure 8. This reduction factor accounts for distance-related events such as interruptions where no heat input occurs, as well as the reduction observed in the IPCF, which exhibits a decrease in the *COF*.

The sigmoid function fit, as shown in Figure 8A,B, provides a close approximation that describes the reduction in *COF* observed in the IPCF. Whether a transition between low and high friction occurs or not, the sigmoid function fits the data. In cases where no transition is detected, a negative transition value is assigned directly by the fitting function. The fitted data are then divided by the maximum *COF* value, which approximates the steady-state condition. The IPCF heat flux reduction factor *β*_COF_ can be determined for both cutting and interruption segments. The steady-state heat flux within the tool–chip interface is multiplied by the reduction factor along the tool movement pathway, accounting for multiple heat cycles. The difference between dry and MQL in Figure 8C is caused by low friction within the IPCF when MQL is applied and appears as low *COF*. This effect is reflected in the heat flux reduction factor that is used later to calculate the transition temperature of the IPCF.

### 3.4. Heat Flux and Temperature Results

**Figure 9:**
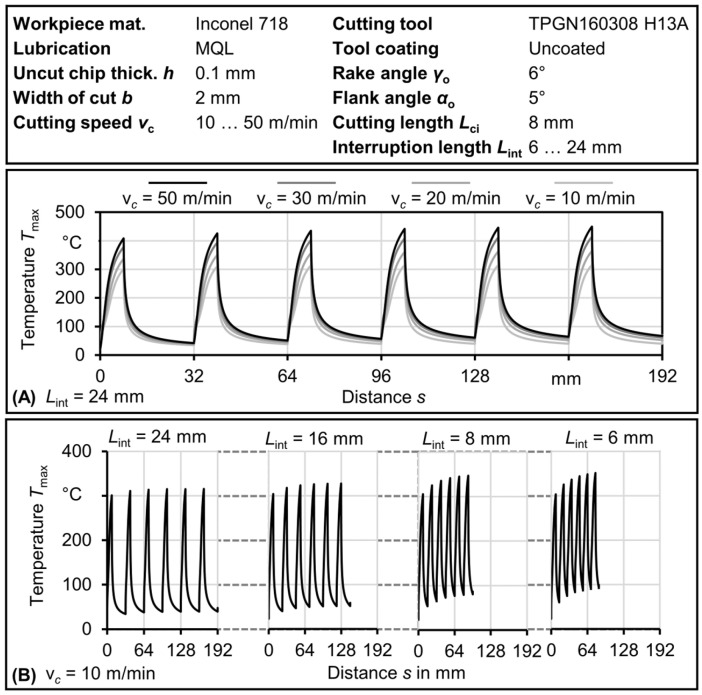
Maximum rake temperature at the rake face obtained from the model in interrupted cutting for Inconel 718: (**A**) at different speeds for *L*_int_ = 24 mm; (**B**) at different interruption lengths at *v*_c_ = 10 m/min.

This section presents the temperature within the tool, resulting from the determined heat flux. Figure 9 displays selected temperature results obtained from the model, specifically showing the maximum temperature on the rake face.

Figure 9A demonstrates that higher cutting speeds result in higher maximum temperatures at the tool–chip contact, as expected, due to the increased heat flux. However, the heat partition ratio may limit its influence on the tool temperature, as only a small amount of heat enters the tool at high speeds. During cutting, the temperature gradually rises due to the heat supplied by the cutting process, resulting from shearing and friction. When cutting is interrupted, the temperature increase halts and a reduction in temperature occurs. Figure 9B shows that shorter interruption lengths lead to higher minimum and maximum temperatures, as the time available for cooling the tool becomes shorter. The temperature change associated with different interruption lengths suggests a potential impact of temperature on the reduced tool–chip contact in the lubricated IPCF, where temperature and interruption time become crucial factors. At low speeds and longer interruption lengths, the reduced contact in the IPCF becomes more noticeable between the dry and MQL cases. Figure 9 reveals that the reduced tool–chip contact friction in the IPCF appears to slow down the temperature increase during cutting.

**Figure 10:**
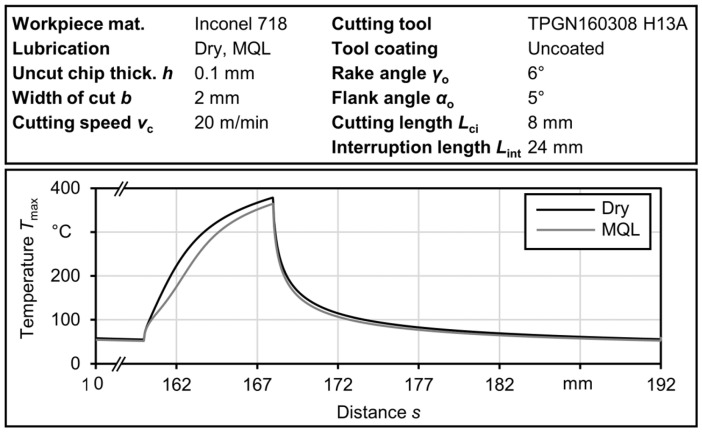
Effect of MQL on maximum rake temperature for Inconel 718.

Figure 10 illustrates a single heating cycle, emphasizing the impact of MQL on the maximum temperature at the tool–chip interface. The heat flux reduction factor, *β*_COF_, plays an important role in reducing the rate of temperature increase at the beginning of the cutting process. This factor reflects the impact of the low apparent coefficient of friction within the IPCF and determines the extent to which a reduction in steady-state heat flux can be achieved. The decrease in heat flux at the start of the cut affects the final temperature before interruption, resulting in a lower maximum temperature achieved using MQL compared to the dry condition.

**Figure 11:**
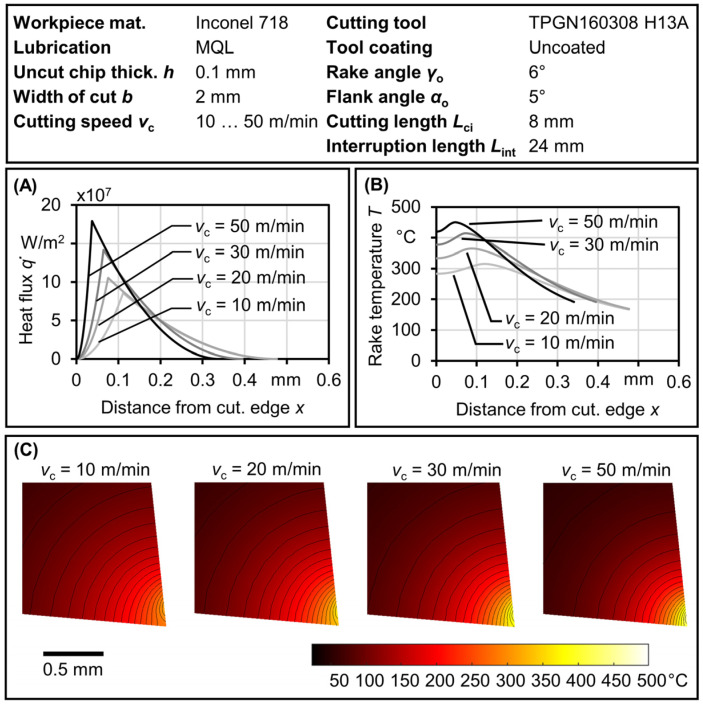
Heat flux and tool temperature for Inconel 718. (**A**) Heat flux along tool–chip contact length at steady state. (**B**) Temperature along tool–chip contact length before interruption. (**C**) Temperature field within the tool at different cutting speeds occurring before interruption.

Figure 11A illustrates the total heat flux along the tool–chip contact length in the steady-state condition, $\dot{q}$(x), for different cutting speeds and lubrication conditions. The heat flux, denoted as $\dot{q}$(x), undergoes reduction via the heat partition ratio, *R*_2_. A fraction of this flux passes through the chip, while the remaining part (1 – *R*_2_) is transferred into the tool. Additionally, the heat reducing factor, *β*_COF_, allows for a non-uniform heat flux that depends on the tool’s position within the IPCF. The non-uniform distribution of heat flux across the tool–chip contact length results in a non-uniform temperature distribution along the contact area. The highest temperature occurs at a specific distance from the cutting edge, as illustrated in Figure 11B. The temperature field within the tool at these maximum values is shown in Figure 11C for different cutting speeds using MQL.

The heat flux illustrated in Figure 11A spans a finite length along the tool–chip contact surface. The end point of this flux and the position of its peak value (found at the end of the sticking zone) impact both the temperature distribution along the contact and within the tool, as demonstrated in Figure 11B. High temperatures are reached at high cutting speeds, although the temperature field within the tool may vary, as seen in Figure 11C. At low cutting speeds, a lower maximum temperature is observed, but with an extended temperature field due to the longer tool–chip contact length.

**Figure 12:**
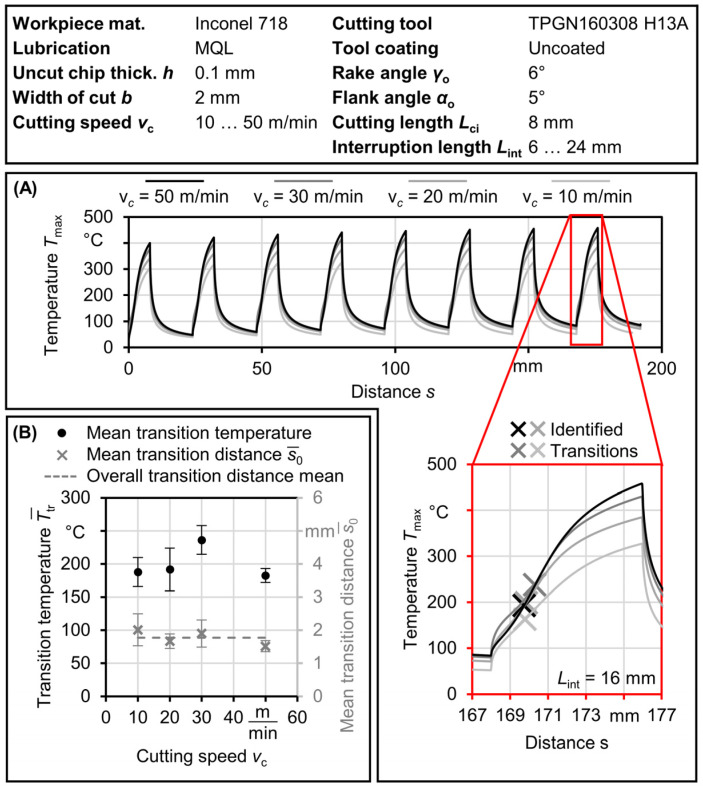
Identification of transition temperatures. (**A**) Example of transition temperature identification. (**B**) Transition temperature and distance results.

The temperature analysis discussed so far raises an important question regarding the existence of a transition temperature for the IPCF and its quantification. Figure 12 attempts to provide more detail on this aspect.

As previously shown in Figure 8A,B, a sigmoid function was utilized to fit the data of the apparent coefficient of friction (*COF*). The transition distance, denoted as *s*_0_, was determined as the value at the sigmoid’s midpoint. In Figure 12, the identified transition distance was employed to find the transition temperature at different cutting speeds. It should be noted that the transition distance is only valid for cases with MQL; the dry cases either exhibit negative values or very small values that were not considered to be transition points. An example illustrating how the transition temperature is identified is presented in Figure 12A. A summary of the transition results is provided in Figure 12B. The transition distance, *s*_0_, appears to remain constant across different cutting speeds at an average value of $\bar{s_{0}}$ = 1.8 mm, and the corresponding transition temperature, *T*_tr_, falls within a limited range with an average of $\bar{T_{\mathrm{tr}}}$ = 200 °C. The relationship between transition distance and transition temperature and the minimal effect of cutting speed characterizes the transition of the IPCF into the steady state.

## 4. Discussion and Conclusions

Transient thermal modeling of the IPCF in interrupted machining enables a deeper understanding of the working mechanism of the IPCF. The heat flux in interrupted machining is cyclic, reaching zero during interruption and having a reduced value at the IPCF. Consequently, the temperature becomes cyclic with transient behavior that is always present, regardless of the contact condition. In the IPCF, the transient temperature rise can be delayed, resulting in a reduced maximum temperature before the next interruption interval. However, for this additional reduction in temperature to be observed, the transition associated with the IPCF between low and high friction must occur. This transition was only observed when effective application of a lubricant was achieved.

An important observation is that the transition distance, as obtained from fitting a sigmoid function, lies within a very narrow range. A similar observation holds true for the corresponding transition temperature. It was found that the transition distance was less than a couple of millimeters, and the corresponding mean transition temperature was about 200 °C.

Uncertainties related to the accuracy of heat flux determination might also affect the model results. In particular, the calculation of sticking length and the assumption that the pressure distribution exponent *ζ* and chip velocity distribution exponent *ω* are constants at variable cutting conditions might contribute to increased uncertainty in accurately determining tool temperature. Further experimental investigations are still required to validate the transient temperature rise on the rake face in real-time, which is still one of the major limitations in machining research.

## Figures and Tables

**Table 1:** Temperature-dependent thermal properties and density of tool and workpiece materials.

Material	Property	Equation or Value
Tool WC/6Co adapted from Spriggs et al. [39]	Thermal cond. in W/mK	$k\left(T{}^{\circ}C\right)=3\times 10^{-5\;}T^{2}-0.0715\;T+100.25$
	Specific heat in J/kgK	$c\left(T{}^{\circ}C\right)=-6\times 10^{-5}\;T^{2}+0.125\;T+213.07$
	Density in kg/m^3^	15,160 *
Inconel 718 adapted from Sweet et al. [40]	Thermal cond. in W/mK	k(T°C)=0.017 T+10.73
	Specific heat in J/kgK	$c\left(T{}^{\circ}C\right)=-2.9\times 10^{-4\;}T^{2}+0.44\;T+330$
	Ther. diffusivity in m^2^/s	$K\left(T{}^{\circ}C\right)=2.83\times 10^{-9}\;T+2.82\times 10^{-6}$
	Density in kg/m^3^	8221

* Measured.

## Data Availability

Dataset available on request from the authors.
